# Studying O_2_ pathways in [NiFe]- and [NiFeSe]-hydrogenases

**DOI:** 10.1038/s41598-020-67494-5

**Published:** 2020-06-29

**Authors:** Tiago M. Barbosa, Carla S. A. Baltazar, Davide R. Cruz, Diana Lousa, Cláudio M. Soares

**Affiliations:** ITQB NOVA, Instituto de Tecnologia Química e Biológica António Xavier, Universidade Nova de Lisboa, Av. da República, 2780-157 Oeiras, Portugal

**Keywords:** Bioenergetics, Computational biophysics, Biophysics, Molecular biophysics, Molecular conformation, Thermodynamics, Computational biology and bioinformatics, Computational models, Protein analysis, Protein structure predictions, Structural biology, Molecular modelling

## Abstract

Hydrogenases are efficient biocatalysts for H_2_ production and oxidation with various potential biotechnological applications.[NiFe]-class hydrogenases are highly active in both production and oxidation processes—albeit primarily biased to the latter—but suffer from being sensitive to O_2_.[NiFeSe] hydrogenases are a subclass of [NiFe] hydrogenases with, usually, an increased insensitivity to aerobic environments. In this study we aim to understand the structural causes of the low sensitivity of a [NiFeSe]-hydrogenase, when compared with a [NiFe] class enzyme, by studying the diffusion of O_2_. To unravel the differences between the two enzymes, we used computational methods comprising Molecular Dynamics simulations with explicit O_2_ and Implicit Ligand Sampling methodologies. With the latter, we were able to map the free energy landscapes for O_2_ permeation in both enzymes. We derived pathways from these energy landscapes and selected the kinetically more relevant ones with reactive flux analysis using transition path theory. These studies evidence the existence of quite different pathways in both enzymes and predict a lower permeation efficiency for O_2_ in the case of the [NiFeSe]-hydrogenase when compared with the [NiFe] enzyme. These differences can explain the experimentally observed lower inhibition by O_2_ on [NiFeSe]-hydrogenases, when compared with [NiFe]-hydrogenases. A comprehensive map of the residues lining the most important O_2_ pathways in both enzymes is also presented.

## Introduction

Hydrogenases are metalloenzymes that catalyse the reaction of H_2_ ⇋ 2H^+^ + 2e^−^^1–4^. Functioning at a high turnover frequency, they are considered the most efficient noble-metal free H_2_ production and oxidation catalysts, being at least as effective as economically expensive platinum based catalysts^5–7^. Their applications are many, ranging from fuel cells to electro- and photocatalysis^5–7^. Studying their catalytic mechanisms is very important for making H_2_ an economically viable, carbon-free alternative to current energy sources. Most hydrogenases are sensitive to O_2_, which is one of the major problems for their use in large scale applications^3^. Therefore, studying the behaviour of O_2_ inside the structure can be extremely valuable and may open new avenues in their engineering.

Hydrogenase nomenclature is based on the composition of their bimetallic active centre, with [NiFe]- and [FeFe]-hydrogenases being the two most common hydrogenases in nature^2^.[FeFe]-hydrogenases are generally irreversibly inactivated and damaged by O_2_^8^, while the [NiFe]-class shows a more diverse behaviour towards exposure, being typically reversible^9,10^. Reflecting on the heterogeneity and variety of different hydrogenases a classification was proposed for the known enzymes, taking into account the composition of the active centres, physiological function and cellular location^11^. In this classification four groups of [NiFe]-hydrogenases exist—being divided by function and location. The present study focuses on two hydrogenases belonging to group 1, membrane-bound H_2_ uptaking [NiFe]-hydrogenases: A group 1a *Desulfubrio gigas* [NiFe]-hydrogenase and a group 1b *Desulfubrio vulgaris* [NiFeSe]-hydrogenase.

There are several common features among the group 1 hydrogenases. They are ~ 100 kDa, periplasmatic, multi-subunited proteins, which are often membrane bound and very sensitive to temperature and pH alterations. These features define what we call ‘standard’ [NiFe] hydrogenases, which are normally oxygen sensitive group 1 hydrogenases. However, ‘non-standard’ hydrogenases exist and display very different and interesting characteristics, ranging from oxygen insensitivity (even in environmental conditions) to thermostability. These enzymes often display active centres similar to the ‘standard’ ones, which raises the hypothesis that oxygen insensitivity may indeed come from the surrounding structure of each centre. Most [NiFe] hydrogenases are primarily hydrogen catalysts, which supports their biotechnological interest, for instance in fuel cells^10^.

The inactivation of standard [NiFe] hydrogenases occurs by the formation of a mixture of two inactive states (Ni–A and Ni–B) in the active centre^12,13^. While in the inactive states, the Ni ion is in a Ni(III) oxidation state and a bridging hydroxo ligand is present between the Ni and Fe ions^14^; other modifications also contribute to the inhibition, as the oxidation of the sulfur ligands^4,9,14–18^, and the main difference between the Ni–A and Ni–B states has been proposed to be an S-oxygenated bridging cysteine in the former when compared with the latter^14^.

**Figure 1:**
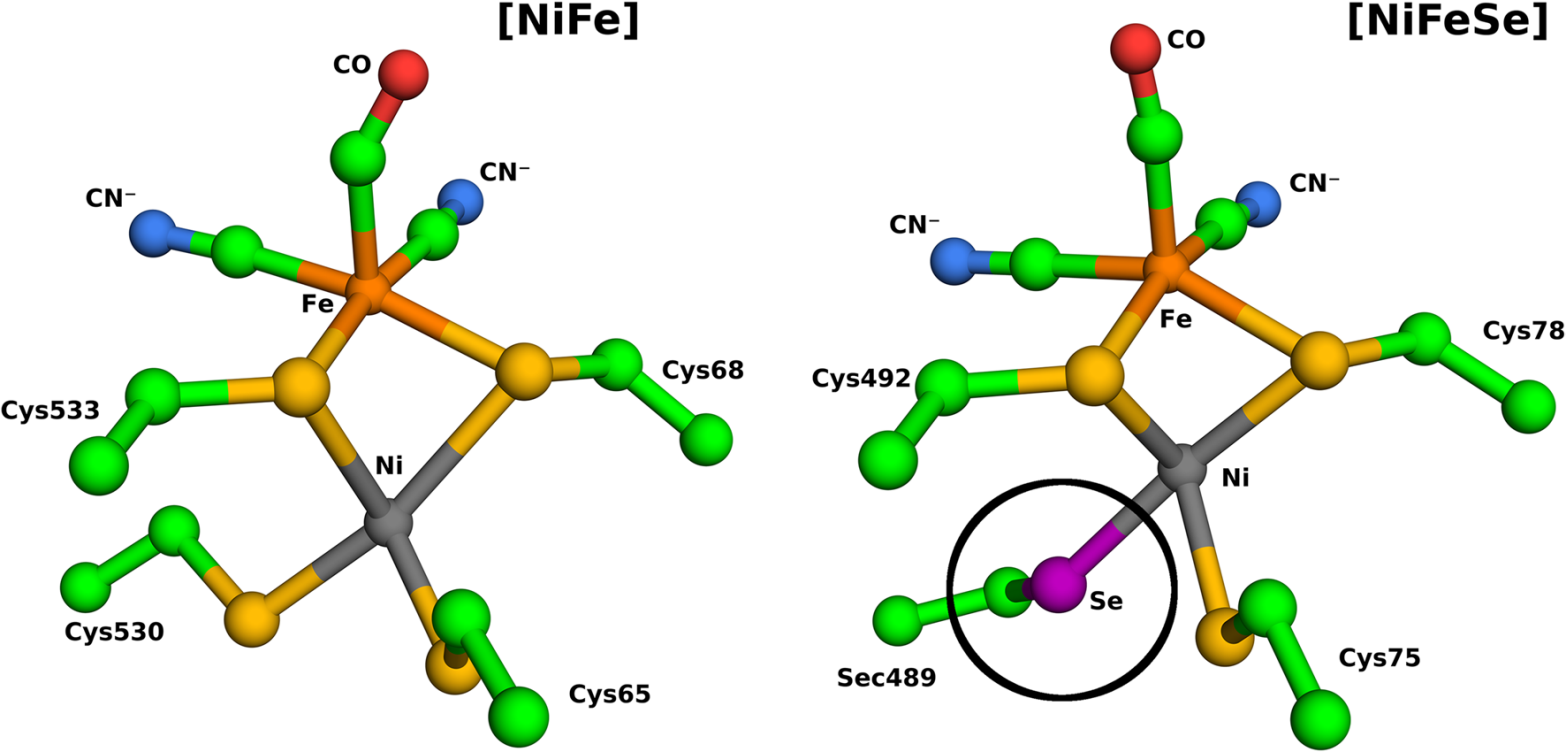
Molecular representation of the active sites of [NiFe]- and [NiFeSe]-hydrogenases, containing their protein (cysteine and selenocysteine residue, truncated at the C-alpha) and non-protein ligands (CO and CN^−^). The selenocysteine residue is highlighted by a black circle). The color coding of the different atoms is the following: carbon: green; oxygen: red; nitrogen: blue; sulphur: yellow; selenium: magenta; nickel: dark gray; iron: orange. Labels are placed to identify the metals, protein residues and ligands.

[NiFeSe]-hydrogenases are a subclass of [NiFe]-hydrogenases, which are mainly characterized by having a selenocysteine coordinating the Nickel in the active site^19^ (Fig. 1). They display, particularly in the case of the hydrogenase we are studying, very interesting properties, such as high catalytic activities and shorter reactivation times when exposed to O_2_, when compared to [NiFe]-hydrogenases, making them more suited to biotechnological applications^19,20^. Recovery time from the oxidised states is remarkably different, as [NiFeSe]-hydrogenase is extremely fast, while standard [NiFe] can take several hours^21^.

Most [NiFeSe] hydrogenases are isolated from organisms that inhabit in anaerobic habitats^22^, therefore not having any evolutionary pressure to establish O_2_ protection mechanisms.

Structurally, the two hydrogenases enzymes that we study here are almost identical, being comprised by of two subunits. The active site lies deep inside the large subunit, while the small subunit generally contains three iron–sulphur clusters in a wire like formation, forming an electron transfer chain between the active site and the enzyme surface. The exact cluster composition differs: in the O_2_ insensitive [NiFeSe]-hydrogenase the iron–sulfur clusters are all [4Fe4S], while in [NiFe]-hydrogenase there are two [4Fe4S] and one [3Fe4S]^4,23–25^.

Several structural features of the [NiFeSe] hydrogenase have been evidenced to explain its catalytic prowess: the “cage effect” of the protein structure surrounding the active site^26^, differences in residues comprising proton transfer pathways and H_2_ channels^27^ and the nature of the selenocysteine complex. The complex has been suggested^24^ to have a severe influence on the O_2_ sensitivity of the [NiFeSe]-hydrogenase, both by promoting the rapid recovery from O_2_ damage as well for increasing H_2_ production^28–30^. Other factors, such as the access of O_2_ to the active site, may also play a role in the unique feature of [NiFeSe] hydrogenases.

Determining the O_2_ paths via experimental methods is very challenging, as O_2_ is very mobile, has a low electron count and weak interaction with amino acids^31^. Therefore, computational studies on the subject are then a valuable way to propose pathways by directly observing a representation of the nature of the phenomenon in an atomic level. MD simulation studies, and techniques using this type of simulation data, are particularly promising, since they can go beyond the “static” picture conveyed by many experimental structural data (which often correspond to averages over molecular configurations and time), by sampling many discrete configurational states, providing a molecular ensemble of states that can be analysed in full detail. Additionally, these methodologies, coupled with special techniques, can provide energetic estimates of micro- and macro-state transitions of experimentally correlatable phenomena, such as O_2_ permeation studied here.

Permeation pathways for entrance of H_2_ in [NiFe]- and [NiFeSe]-hydrogenases have been studied using computational methods by us and other authors^27,32,33^, but the subject of O_2_ permeation has been less studied^31,32^, and, to the best of our knowledge, never studied on a [NiFeSe]-hydrogenase. Therefore, the aim of the present work is to study a [NiFe]- and a [NiFeSe]-hydrogenase to compare their differences in O_2_ internalization, diffusion and protection inside the protein structures, trying to unravel the structural and dynamic differences that might explain the different O_2_-sensitivity. With the present study, we were able to map the free energy landscape for O_2_ permeation on both enzymes and found very marked differences. Analysing these landscapes using probabilistic models has shown evidence for a more defined pathway for O_2_ internalization in [NiFe]-hydrogenase and a more diffuse and less specific set of pathways in [NiFeSe]-hydrogenase.

## Results and discussion

Molecular Dynamics (MD) simulations were performed on a [NiFe]- and a [NiFeSe]-hydrogenase structures (PDB ids 2FRV and 2WPN, respectively). Two sets of simulations were run for each enzyme; the first in water with counter-ions and the second in water, counter-ions and 100 molecules of explicit O_2_. Five trajectories for each set and enzyme were run, each lasting 100 ns (for the simulations in water and counter-ions) or 70 ns (for the simulations in water, counter-ions and O_2_).

The trajectories for both enzymes in water show a stability plateau after about 20–30 ns, as can be seen in Figure S1 of Supplementary material, which displays the c-alpha atomic positions root mean square deviations (RMSD). Additionally, introducing the O_2_ in the system did not compromise this stability.

**Figure 2:**
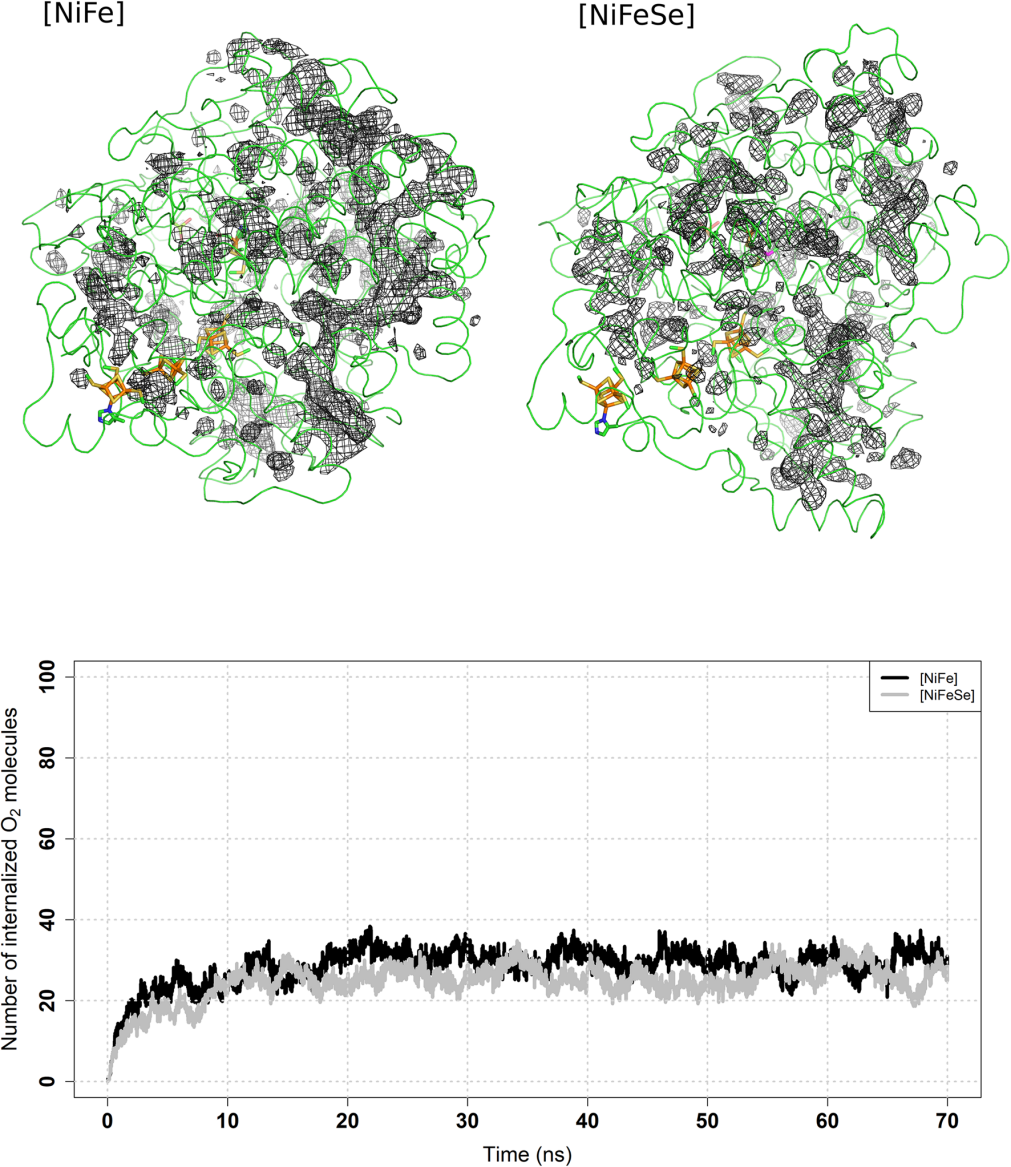
Top—slice of the protein structure with PDF’s represented by wireframe meshes at probability 0.002. PDFs were calculated from 35 to 70 ns. The proteins are represented by a green ribbon while metal centres are represented using sticks. The centres are represented as sticks with the iron atoms coloured light orange, nickel in gray, carbon in green, oxygen in red, nitrogen in blue and sulphur in yellow. Bottom—average number of internalized O_2_ molecules over time.

To illustrate O_2_ internalization we calculated average Probability Density Functions (PDFs) from the five trajectories calculated for each hydrogenase (Fig. 2). The probability maps show similar patterns of internalization on both enzymes, with a main channel, in an approximately perpendicular orientation with the line formed by the three FeS centres leading to the active site (but relatively far from it). There are also diffuse zones of higher probability all around both enzymes and several zones where the probability is not continuous. There are not enough continuous zones of high O_2_ probability near the active centres to be able to define pathways. This is likely due to the insufficient sampling provided by the simulated timescale.

Figure 2 also contains a plot of O_2_ internalization, which shows that the quantity of molecules internalised reaches a plateau at ~ 30 molecules out of the total 100, for both enzymes, and this process is relatively fast (~ 10 ns) in both cases. From this data, we conclude that, within the simulated time scale, both the [NiFe] and the [NiFeSe] enzymes, do not show any differences in the capacity to internalise and hold molecular oxygen.

Interesting as these results may be, it is also clear that the sampling obtained in the time scale of these simulations does not allow to adequately find clear paths for molecular oxygen permeation, up until the active site zone. This is in contrast with our previous experience with molecular hydrogen in these hydrogenases, which rapidly reaches the active site^26,27,34^ and this is certainly due to the significant larger size of molecular oxygen, when compared with molecular hydrogen. We have previously observed this type of situation on oxygen metabolising enzymes^35,36^ and the solution we used in these cases was to resort to Implicit Ligand Sampling (ILS), which can explore higher energy zones in the permeation free energy surface. This was the route we decided to follow in the present work, and use the oxygen free trajectories of the enzymes in water to infer about the free energy surface of molecular oxygen placement, in the whole space of the hydrogenases.

**Figure 3:**
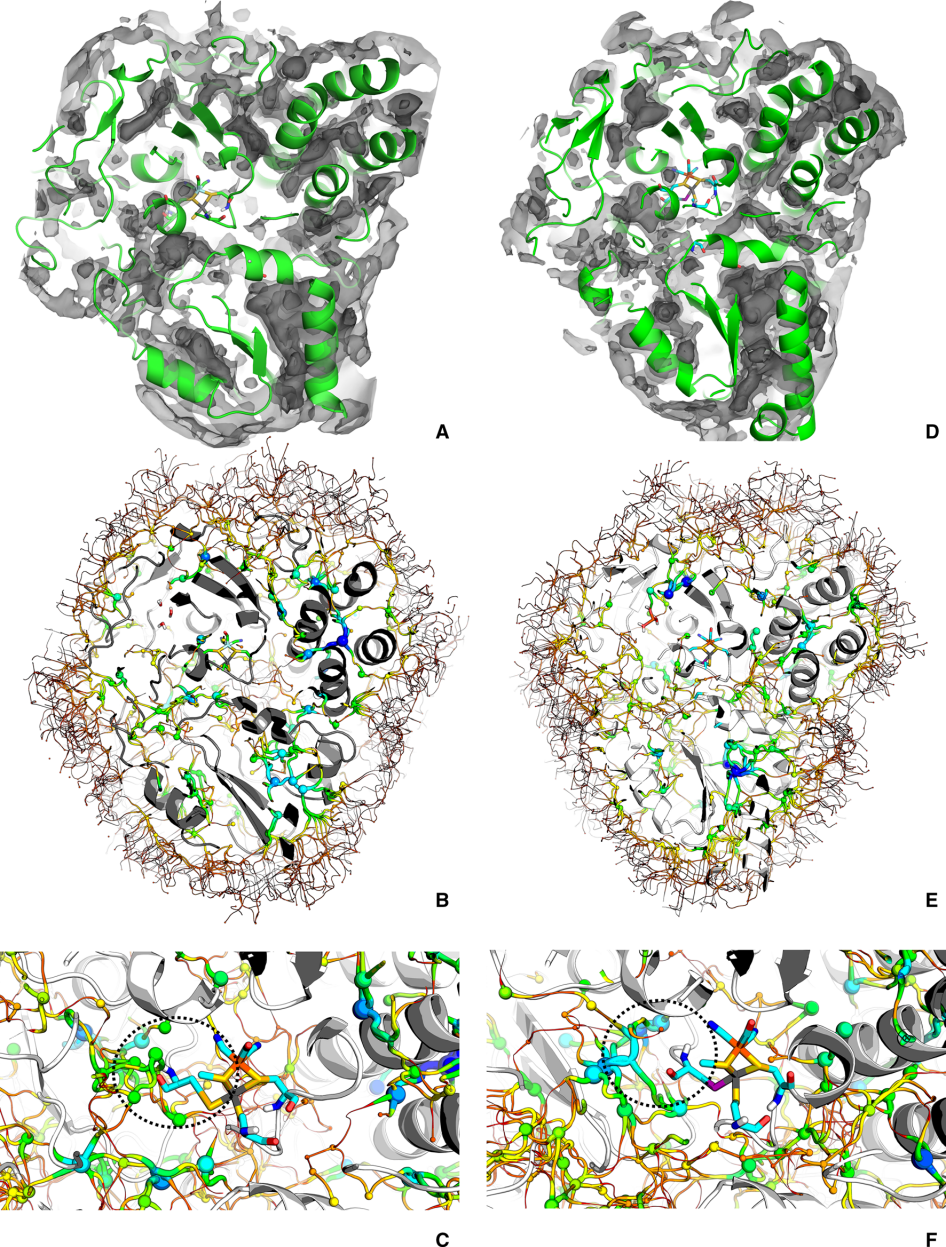
(**A**–**C)** [NiFe]-hydrogenase; (**D**–**F)** -[NiFeSe]-hydrogenase; (**A**,**D)** ILS isosurfaces—energy cut-offs of − 1, − 5, − 10 kJ/mol from lighter to darker grey. (**B**,**E)** overview of the pathway tessellation—minima are represented by spheres and the pathways by cartoon traces. Pathway energy is inversely proportional to the thickness. Both are colour coded from lower (− 16 kJ/mo^1^) to higher (2 kJ/mol) energy—blue, yellow, green, orange, red) respectively. (**C**,**F)** The selenocysteine and corresponding cysteine are evidenced by the dotted black circle. The conformations of the proteins were selected from frames within the equilibrate part of a single trajectory for each enzyme.

By applying the ILS methodology to a trajectory, O_2_ was forced in the whole space of both enzymes, mapping even the deeper structural layers. This comprehensive analysis allowed a detailed examination of the free energy interaction landscape between molecular oxygen and the whole protein, including the active centre zone. This interaction free energy includes the enzyme’s natural conformational variability, as sampled by the five replicate trajectories along the selected simulated time (the last 10 ns of these trajectories). Note that what is averaged here is the interaction free energy between molecular oxygen and the protein, since the ILS calculations are performed in every frame selected from the five replicate trajectories and not on their average conformations. Figure 3A, D displays the results of this method applied to the five trajectories of the [NiFe]- and [NiFeSe]-hydrogenases, respectively.

These results are consistent with the MD simulations with molecular oxygen, as the lower energy zones are roughly similar with the higher PDFs evidenced on Fig. 2. However, in the case of the ILS results of Fig. 3, low probability zones near the active sites can also be defined, evidencing the higher sampling power of ILS, when compared with the MD simulations with explicit O_2_.

Panels B and E of Fig. 3 show that the tessellation pathways are extremely intricate; a detailed visual observation (results not shown) evidences multiple low energy paths coming from the outside of both hydrogenases. These multiple low free energy basins occur in regions correlatable with the entry pathways found in MD with explicit O_2_.

Figure 3 also contains (panels C and F) the ILS pathways found near the active centres, represented by the minima (as spheres) and the paths between minima (as cartoon traces). By analysing these two landscapes near the active centres, it is evident that the [NiFe]-hydrogenase contains more low energy basins near the cysteine that is replaced by a selenocysteine (Sec) in the [NiFeSe] enzyme, the latter being relatively empty of basins in the same location (circled zones in panels C and F of Fig. 3). This is already an indication for the higher difficulty of placing O_2_ near the active site in the [NiFeSe]-hydrogenase, when compared with the [NiFe] counterpart. Part of the reasons for this may lie on the larger size of the selenium of selenocysteine, when compared with the sulphur of cysteine. Therefore, the protein structure and dynamics of the [NiFeSe]-hydrogenase seem to be better adapted to reduce the O_2_ access to the active site, when compared with the [NiFe]-hydrogenase, which can be used to explain the lower O_2_ sensitivity of the former, when compared with the latter. This is interesting and in contrast with our findings for H_2_ permeation^27^, where, using MD simulations, we found higher density for H_2_ in [NiFeSe]-hydrogenase, when compared with the [NiFe]-hydrogenase. We hypothesized that this different H_2_ permeation was the molecular basis to explain the higher catalytic activity towards H_2_ of [NiFeSe]-hydrogenase and its faster reactivation.

With flux analysis using transition path theory (TPT), we can calculate the net flux of O_2_ from the exterior of the protein to the active site. From this overall analysis we determined the flux of O_2_ to the active site of both hydrogenases, and the values are 5.28 × 10^−5^ and 1.20 × 10^−5^ for the [NiFe]-hydrogenase and the [NiFeSe]-hydrogenase, respectively. With this we put a number on the visual analysis present in Fig. 3, clearly showing the higher capacity of [NiFe]-hydrogenase to permeate O_2_, when compared with the [NiFeSe]-hydrogenase. As said above, this correlates well with the lower O_2_ sensitivity of [NiFeSe]-hydrogenase.

**Figure 4:**
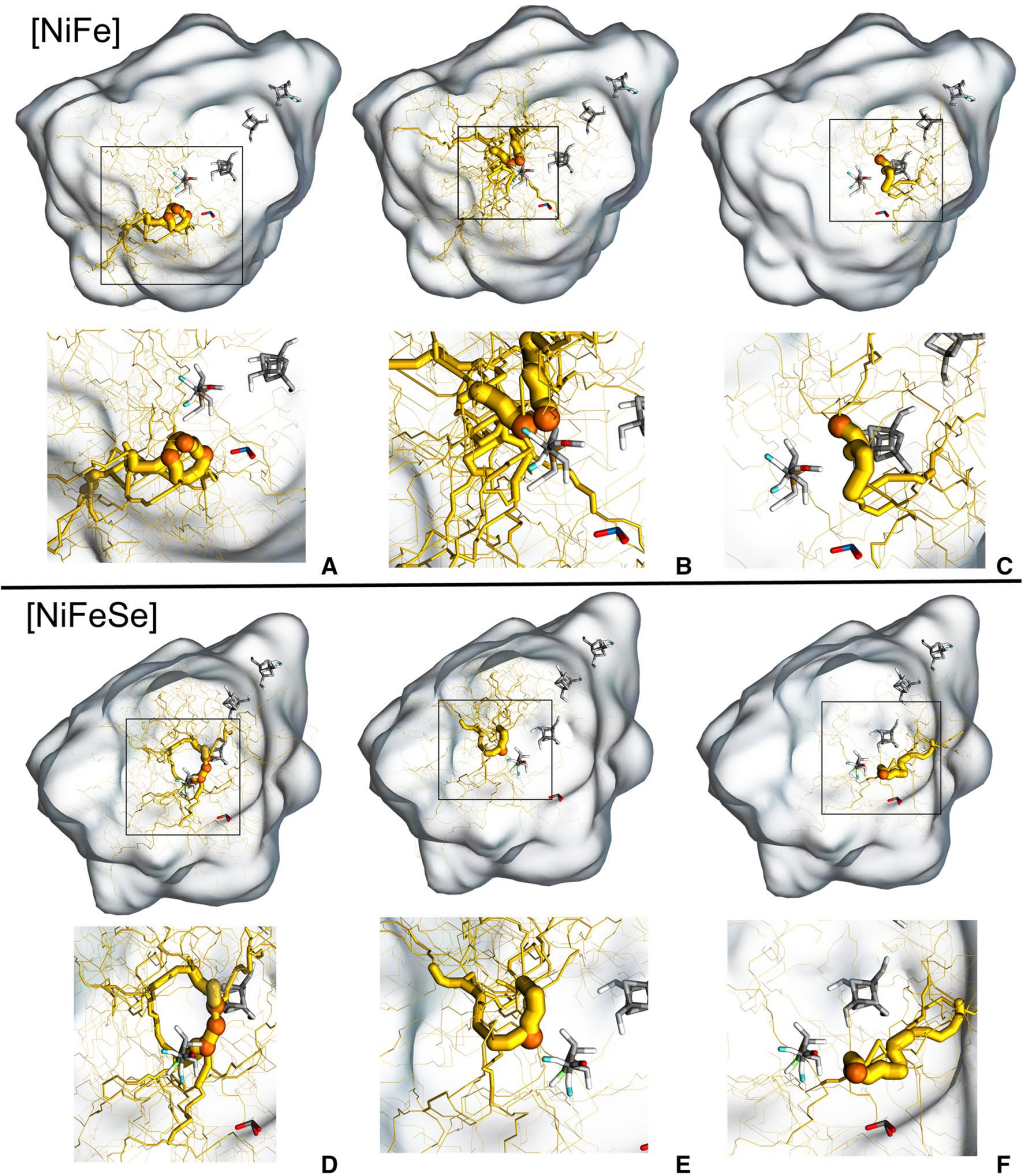
Main fluxes found by reactive flux analysis of the ILS pathways targeting basins near the active centre. The net flux is represented by the yellow trace (thickness proportional to the normalized flux—non comparable between different paths); target basins are represented by the orange spheres. Pathways were denominated as: (**A)** NF-A; (**B)** NF-B; (**C)** NF-C; (**D)** NFS-A; (**E)** NFS–B; (**F**) NFS-C. Slight different orientations of the two hydrogenases were chosen to optimise the visualisation of the pathways and residues.

There are a number of pathways contributing to the overall flux towards the active site of both hydrogenases. These are displayed in Fig. 4 and quantified on Table 1, where the final energy basins are identified. We decided to highlight sets of pathways instead of individual ones, since these appear in interconnected clusters. Note also that the sum of the fluxes of the pathways on each enzyme does not correspond to the complete flux calculated, since these pathways can, sometimes, use parts of the other pathways, having common sub pathways among them. Figure 4 shows the paths on the whole protein with inset highlights of the active site zone. We selected the reactive pathways with a flux higher than 50% of the highest flux (from basin to basin) for each hydrogenase. Each pathway comprises product basins apparently sharing the same reactive network. Table 1 describes the net flux values and the pathway selection.

**Table 1:** Grouping of reactive pathways per product basin and respective nomenclature. Percentages were calculated from the sum of all the fluxes for each enzyme. Note that percentages (% of sum) cannot be compared between different enzymes, since these are calculated using the data of each enzyme.

Enzyme	Pathway designation	Product basin	Flux	% of sum
[NiFe]	NF-A	NFA-1	4.06 × 10^−5^	36.03
		NFA-2	3.09 × 10^−5^	27.37
		NFA-3	2.64 × 10^−5^	23.36
	NF-B	NFB-1	2.11 × 10^−6^	1.87
		NFB-2	1.10 × 10^−5^	9.76
	NF-C	NFC-1	1.82 × 10^−6^	1.61
[NiFeSe]	NFS-A	NFSA-1	2.71 × 10^−6^	17.97
		NFSA-2	1.80 × 10^−6^	11.98
		NFSA-3	1.88 × 10^−6^	12.51
	NFS-B	NFSB-1	6.95 × 10^−6^	46.12
	NFS-C	NFSC-1	1.72 × 10^−6^	11.41

Several entrance pathways were found in both hydrogenases, suggesting the presence of multiple entry points on the protein surface. The reactive pathways are remarkably different, converging to different points near the centres, suggesting multiple inactivation mechanisms and kinetics for each. The existence of multiple pathways for O_2_ permeation have been evidenced before by previous modelling^32^ and combined experimental and modelling^31^ works on related [NiFe]-hydrogenases, and seems to be a characteristic of these systems, as further evidenced by the results we present here on Fig. 4 and Table 1.

Overall, this analysis provides evidence for a main pathway towards the active centre in the [NiFe]-hydrogenase (NF-A), which has dominant flux values, contrasting with the several representative pathways in the case of the [NiFeSe]-hydrogenase. Nevertheless, NFS-B is dominant in [NiFeSe]-hydrogenase. The values of the fluxes are considerably higher for most of the [NiFe]-hydrogenase target basins, when compared with the [NiFeSe] enzyme, which correlates well with the higher value of the total flux found for the former.

Path NF-A of [NiFe]-hydrogenase comprises three target basins sharing the same network. The pathway converges directly to the Ni coordinating Cys 530L, which is replaced by a Sec in [NiFeSe]-hydrogenases. Interestingly, oxidation of this cysteine has been experimentally found in a related [NiFe]-hydrogenase from *Desulfovibrio vulgaris* Miyazaki^15,37^. This pathway has no representation in the [NiFeSe]-hydrogenase and accounts for a large part of the flux, suggesting that it may be the one of the main inactivation spots in [NiFe] enzymes of this group. The presence of selenium in the [NiFeSe]-hydrogenase may also influence inactivation, as it was suggested in previous research^28^.

As for the NF-B pathway of [NiFe]-hydrogenase, its target basins are located near the Fe ion of [NiFe]-hydrogenase, and have similarly located and contiguous basins in the [NiFeSe]-hydrogenase (path NFS-B), suggesting that these two pathways are conserved among the two hydrogenases. Both pathways actually converge in the direction to the hydroxo bridge between the Ni and Fe ions, which is present in the crystal structure of *D. gigas* (but not in the state simulated here—Ni-SI_a_ state). Therefore NF-B and NFS-B basins may be reflecting an inactivation path for O_2_, with end positions (but not the whole path) conserved between the two hydrogenases.

NF-C from [NiFe]-hydrogenase converges to a zone somewhat near the active centre metal coordinating residues Cys 65L and Cys 68L, and has a very low flux. This pathway has correspondence with the NFS-C of the [NiFeSe]-hydrogenase pathway, albeit with a relatively distant convergence spot near Cys 492L.

Similarly to NF-A, pathway NFS-A is also comprised of three product basins, with their respective reactive networks, and converges to an intermediate location between the proximal FeS centre and the active centre in the [NiFeSe]-hydrogenase near Cys 75L, which was already found to be oxidated^24^. This is supported by previous works where [NiFeSe] hydrogenases have displayed sulfinate formation at the non-selenocysteines and oxygenation of the proximal [4Fe4S] cluster^12,29^.

These findings suggest that the preferred pathways for O_2_ differ in both enzymes, possibly determining the inactivation mechanism, as the active site of the [NiFeSe]-hydrogenases is less exposed to O_2_. These differences might be related with specific aminoacid residue changes between the two enzymes; for instance while NF-B in [NiFe]-hydrogenase and NFS-B in [NiFeSe]-hydrogenase converge to the same place in between the two metals, NFS-B is confurcated (resulting from convergence of two convergent pathways), while NF-B is not. The explanation for this may be rather complex, but we notice one residue difference related with this confurcation; Asp107L in [NiFe]-hydrogenase (highly conserved in this group), which is replaced by Ser117L in [NiFeSe]-hydrogenase.

The fact that neither basins nor pathways are present near the selenocysteine (as opposed to the same space of the [NiFe] Cys 530L) suggests that the Sec or the surrounding environment may also have a role in the protection of the [NiFeSe] hydrogenase’s centre.

**Figure 5:**
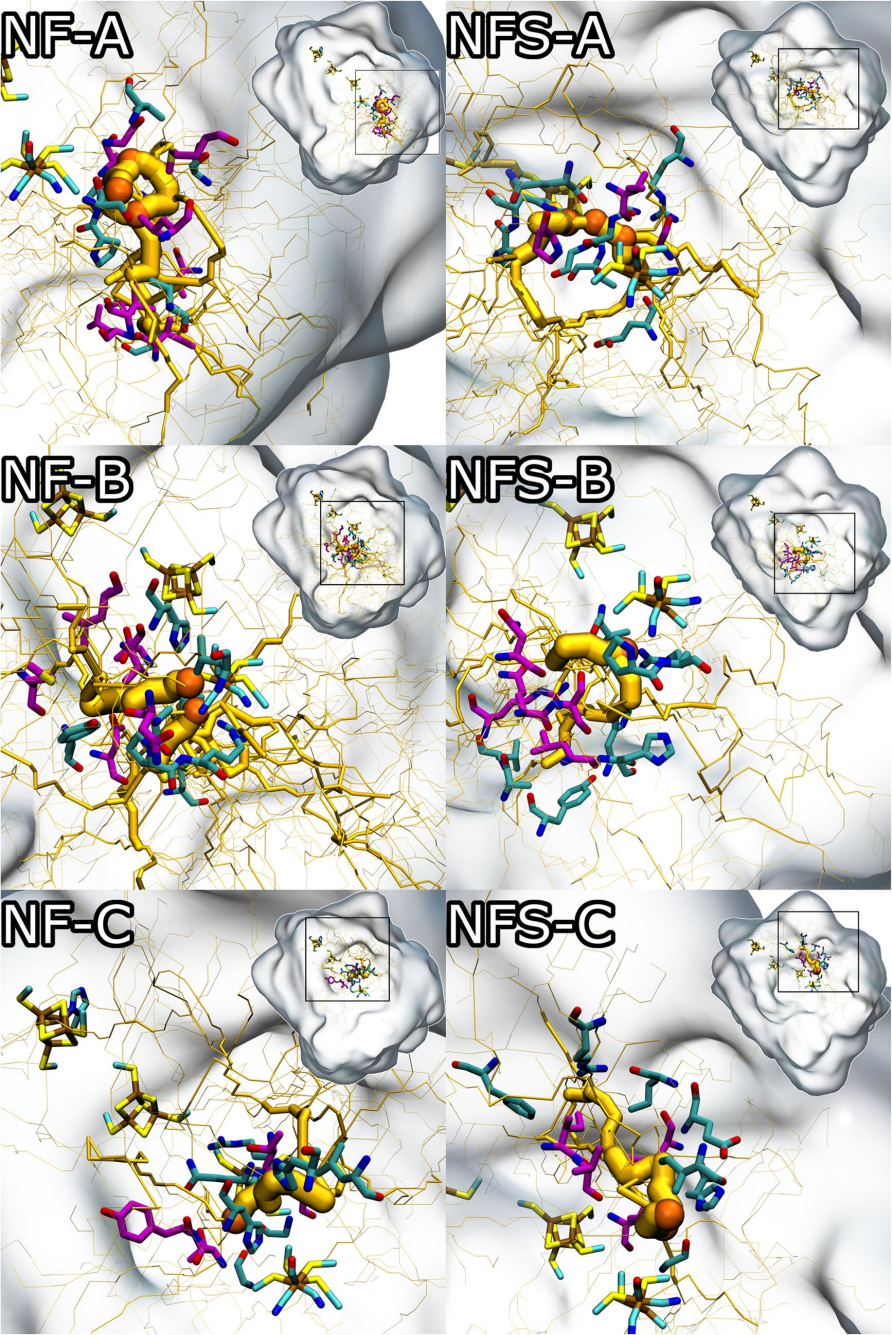
Representation of the residue conservation near the O_2_ pathways. The residues at van der Waals distance (considering the Se:O_2_ distance) from the pathways are selected. Pathways are arranged in the same orientation as Fig. 3, and non-conserved residues are coloured with magenta carbons, while conserved ones are coloured cyan. Slightly different orientations of the two hydrogenases were chosen to optimise the visualisation of the pathways and residues.

To illustrate the differences on the hydrogenase’s O_2_ pathways, we identify all residues at a van der Waals distance of the highest fluxes (higher than one half of the maximum flux the pathway) and mapped them on Fig. 5. The corresponding residue of the other hydrogenase was also selected by aligning the two structures to check for conservation between both hydrogenases (Supplementary material—tables S3-S4, third column).

Figure 5 shows the intricate networks of residues that line the O_2_ permeation pathways and the results contained in tables S3 and S4 (Supplementary material) evidence different degrees of conservation of these residues (note that some residues line more than one pathway). This conservation can be first analysed among the two hydrogenases studied here (third column in tables S3-S4), and secondly among the homologues of each hydrogenase, using the *ConSurf* server^38^ (forth column in tables S3-S4). *ConSurf* provides a measure of sequence conservation within a protein family, using close homologue sequences as a proxy for this protein family.

We established before that, in general, the O_2_ permeation pathways are not the same among the two hydrogenases. However, to different degrees, some of the residues comprising these pathways are the same in the two enzymes. Additionally, these same residues are, in many cases, conserved (but with notable exceptions) within the particular family of a given hydrogenase (we use the ConSurf Color Score as a proxy for the family). Therefore, it is not very likely that these conserved residues can constitute, in isolation, the basis for the different permeation characteristics of the two hydrogenases. On the other hand, the residues that are different among the two hydrogenases, but are conserved in the family of hydrogenases containing a given pathway, are much more interesting to highlight. Analysing residues in the [NiFe]-hydrogenase with a ConSurf Color Score of 9 (corresponding to maximum conservation), we identify Thr 69L and Asp 107L in NF-B and Thr 69L and Val 484L in NF-C. Using the same criteria and looking at the [NiFeSe]-hydrogenase, we can identify Ile 74L, Pro 79L and Gly 491L in NFS-A, Asn 113L, Gln 116L, Ser 117L, Leu 120L and Arg 169L in NFS-B, Ile 74L in NFS-C. These are all residues that may be interesting to look in further computational and experimental mutation studies, which may unravel ways that nature used to evolve different O_2_ permeation features. Actually, in a recent study done in the [NiFeSe]-hydrogenase, one of the residues of NFS-A pathway identified above—Gly 491L—was successfully experimentally mutated by a bulkier alanine residue (the direct [NiFe] counterpart—see table S4) and by a serine residue, leading to decreased O_2_ inhibition, while not affecting H_2_ production, in comparison with the wildtype^39^. This inhibition pathway does not exist in the [NiFe]-hydrogenase, which, according to our results, is mainly inhibited by the NF-A and NF-B paths. Placing a bulkier residue in this position on [NiFeSe]-hydrogenase may eliminate or reduce the NFS-A path, thereby reducing inhibition by O_2_ even further. Another indication that the space occupied by residues within the channels is, in fact, an important factor in the O_2_ inhibition, is the experimental replacement of the active site bulkier selenocysteine residue by a less bulkier cysteine residue in the [NiFeSe]-hydrogenase, which leads to a similar inactivation profile by O_2_ and inactive states similar to [NiFe]-enzymes^28^.

## Conclusions

Using two different approaches, the pathways of O_2_ permeation were comprehensively mapped in two different [NiFe] class hydrogenases structures displaying different O_2_ sensitivities. The methods used here consider, not only the structure, but the dynamic behaviour of the protein structures, allowing for a more realistic analysis that can deal with transient pathways for O_2_ access. ILS in particular allows for a thermodynamic quantification of the O_2_ affinity on the whole protein matrix, which, together with further analysis, allows for predicting the fluxes of O_2_ from the exterior towards the active site of the enzymes.

We found marked differences in the diffusion patterns of both enzymes, being the [NiFe]-hydrogenase more prone for O_2_ access and potential inactivation, when compared with the [NiFeSe]-hydrogenase. Additionally, there is evidence for different mechanisms for O_2_ inactivation of each enzyme, which may help explain the different performances of both in aerobic settings. The pathways for inactivation were also mapped in an atomistic level, which may help understand the structural properties of the focal points of oxygen diffusion. This knowledge may prove useful in future manipulation towards the development of more efficient hydrogen catalysts that are less inhibit by O_2_.

## Methods

### System setup

The X-ray structures of [NiFe] (*D. gigas* PDB ID 3frv)^24^ and [NiFeSe] (*D. vulgaris* PDB ID 2wpn)^25^ hydrogenases were used in this study. Each system was solvated in a rhombic dodecahedral water box using SPC water^40^. A minimum distance of 8 Å between the protein and box walls was imposed. Each system was neutralized with Na^+^ ions to counter act its negative charge. Protonation states were determined through a combination of PB/MC calculations/simulations using MEAD version 2.2.9 and PETIT version 1.6.0 respectively^34,41^ at pH 7.0. These predicted that all lysine and arginine residues were positively charged, while glutamate and aspartate residues were considered negatively charged (but see details on supplementary material for an exception). Details on the Histidine protonation can be found in the supplementary material (Tables S1 and S2).

As for the O_2_ molecule parameters, the model from Cordeiro^42^, which was parameterised to account for the solvation properties of molecular oxygen, both in aqueous as well as non-aqueous environments, was used in this work. As for the oxidation states we considered the Ni-SI_a_ state^43^ for the active [NiFe] centres and the oxidized state for the [4Fe4S] clusters. All the centres are considered to be flexible. More details on the parametrization of the metallic centres can be found in Baltazar et al*.*^27^ and Teixeira et al*.*^33^ for [NiFeSe]-hydrogenase and [NiFe]-hydrogenase, respectively.

### Molecular dynamics simulations

The GROMOS 54A7^44^ forcefield and single point charge (SPC) water model^40^ were used to describe the systems, and GROMACS version 5.0.7^45^ was used to perform all MD simulations. Five 100 ns long simulations in solvent were performed for each system. These simulations were carried out with a constant number of particles, pressure (1 atm—controlled using a semi-isotropic Parrinello-Rahman barostat^46,47^), temperature (300 K—controlled by a V-rescale thermostat^48^) and periodic boundary conditions. Different temperature couplings were applied to protein and solvent + O_2_ atoms using a coupling constant of 0.1 ps. A pressure coupling constant of 1.6 ps was used. All solute bond lengths were constrained with the P-LINCS algorithm^49^ while the SETTLE algorithm^50^ was used for solvent. Equations of motion were integrated with a time-step of 2 fs, with neighbour lists being updated every 40 steps. Electrostatic interactions were treated with the Particle mesh Ewald method^51^ with a real space cut-off at 10 Å and a Fourier grid spacing of 1.2 Å. The Verlet cut-off scheme was selected.

To remove unfavourable atomic contacts, the systems were energy minimized without positional restraints using a combination of steepest descent and Low memory Broyden–Fletcher–Goldfarb–Shanno algorithms^52^. System initialization comprises four 50 ps MD steps with velocities being generated from a Boltzmann distribution at the defined temperature. At the first step, in the *NVT* ensemble, the Berendsen thermostat^53^ was utilized with positional restrains on the C-alpha atoms with force constant of 10,000 kJ/mol Å^2^. Pressure coupling using the Berendsen barostat^53^ was added in the subsequent step with a coupling constant of 3 ps. In the following step all parameters were kept, but the coupling constant was decreased to 2 ps. In the final step all restraints were removed, the pressure coupling constant was reduced to 1.6 ps, with the barostat being altered to Parrinello–Rahman and the thermostat to V-rescale.

A protocol was prepared to study O_2_ diffusion assuring system stability, conformational variety and statistical accuracy. From the solvent only simulations a snapshot of each replicate was retrieved at the 30 ns mark (assuring system stability). 100 water molecules were randomly selected from the outside of the protein structure and substituted by O_2_ molecules. The velocities from the removed water oxygen and one of hydrogen atoms were kept and assigned to the inserted O_2_. The remaining hydrogen atom and its velocity were discarded. 1 ns of equilibration with a smaller timestep (1 fs) was calculated so as the newly introduced molecules stabilize with the solvent (avoiding clashes). The simulations with O_2_ were kept for a further 70 ns amounting to a total of 350 ns of simulation with explicit O_2_ per system.

### MD–O_2_ distribution analysis

The VMD volmap plugin^54^ was utilized calculate probability density functions (PDF’s) of the O_2_ distribution along the MD trajectories with explicit O_2_. A total of 175,000 frames per enzyme, corresponding to the final 35 ns of each trajectory (of the five 70 ns trajectories per enzyme) were used for this calculation, with a grid resolution of 1 Å. We calculated the internalization of O_2_ using a previously implemented and described method^33^. Maps were visualized and images rendered using Pymol (The PyMOL Molecular Graphics System, Version 1.8, Schrödinger, LLC) and VMD^54^.

### Implicit ligand sampling

The implicit ligand sampling^55^ (ILS) method was used to calculate the free energy of transferring O_2_ from pure water to anywhere inside both hydrogenases and surrounding environment. This method allows for studying the whole landscape of molecular oxygen placement, even regions such as the deep lying hydrogenase active site, where explicit molecules of O_2_ have difficulties in reaching within the time scale of the simulation. This methodology uses molecular dynamics simulations of the system without molecular oxygen, in contrast with the previously described simulations.

From the ILS method the potential mean force ($PMF({\varvec{r}})$) of having a diatomic ligand at a position **r** is given by:

1 $$PMF\left( {\varvec{r}} \right) = - k_{b} T\ln \mathop \sum \limits_{m = 1}^{M} \mathop \sum \limits_{k = 1}^{C} \frac{{e^{{ - (k_{b} T)^{ - 1} \Delta E\left( {{\varvec{r}},{\varvec{q}}_{{\varvec{m}}} , \Omega_{k} } \right)}} }}{MC}$$

where $M$ is the number of utilized protein–solvent configurations, $C$ is the number of random orientations of the ligand and $\Delta E({\varvec{r}},{\varvec{q}}_{{\varvec{m}}} , {\Omega }_{k} )$ is the protein–solvent interaction energy in the configuration ${\varvec{q}}_{{\varvec{m}}}$ with the diatomic ligand located at ${\varvec{r}}$ with an orientation ${\Omega }_{k}$. Non-bonded interactions (electrostatic and van der Waals) are accounted by $\Delta E({\varvec{r}},{\varvec{q}}_{{\varvec{m}}} , {\Omega }_{k} )$. In the O_2_ model used^42^, given that it has no partial charges, only van der Waals interactions were considered. For performing these calculations, a modified version^36^ of the GROMACS 4.5.4 Widom TPI algorithm was used to perform ILS^36^. The last 10 ns of the five MD trajectories in water were used (accounting in total for ~ 25,000 configurations for each enzyme), with the configurations being fitted to the C-alpha atoms of the energy minimized structure. Grids of 58 × 62 × 61 Å and 62 × 62 × 63 Å dimensions wad used in the calculations for the [NiFe] and [NiFeSe] structures, respectively. For each grid point, 400 insertions in random positions and orientations (*C* in Eq. 1) per grid cube were made. The results of all calculations were averaged for each system resulting in two discretized scalar fields (3D energy landscapes). These landscapes detail the Gibbs free energy of moving O_2_ from vacuum to a given position of the system, $\Delta G_{vac \to prot } (O_{2} )$. . Finally, as our interest is to study a landscape of the Gibbs-free energy of moving O_2_ from a position in water to a position in the system, $\Delta G_{wat \to prot } (O_{2} )$, we made additional simulations to calculate the free energy of moving O_2_ from the vacuum to water, $\Delta G_{vac \to wat } (O_{2} )$ a subtracting it to every grid point of $\Delta G_{vac \to prot } (O_{2} )$.

To calculate $\Delta G_{vac \to wat } (O_{2} )$ we adopted a method^36^, which takes 10 ns pure water simulations in the NPT ensemble and applies the ILS method to the final 2000 conformations (2 ns). The resulting 3D landscape of this calculation was then averaged over all the grid points resulting in the final $\Delta G_{vac \to wat } (O_{2} )$. The calculated value was of 8.30 kJ/mol for the O_2_ model^42^ used.

### ILS: free energy landscape analysis

ILS details extensively the free energy landscape of both enzymes. Using that information, it is possible to infer low energy pathways of O_2_ inside the structures. To achieve this, a previously implemented method^36^ extending on another previous approach^56^ was adopted. This method starts by linking each grid point to the neighbour grid point of lowest energy (neighbours are defined as the adjacent 26 grid points forming a 3 × 3 × 3 cube around it) until a local minimum is found. All grid points ‘falling’ to the same minima are grouped into sets and classified as basins. After the classification, the algorithm identifies the lowest energy points within the boundaries between each pair of neighbouring basins—the saddle points. A network of paths between all energy minima of the landscape can then be constructed using the steepest-descent paths from the saddle points to the minima.

### O_2_ diffusion kinetics modelling

ILS provides an exhaustive sampling over the energy landscape of the whole system (including high-energy regions) representing a suitable model for a kinetic analysis. In addition, classifying the energy landscape into basins provides a division of the landscape into macrostates. Considering these basins as belonging to the state space of O_2_ diffusion inside the two hydrogenases a Markov process describing the time-discrete evolution of the system in the state space can be constructed. The construction of the representative model relies on calculating a tition probability matrix where each element $$T_{ij} (\Delta t)$$ corresponds to the probability of transition to basin/state $$j$$ after a time $$\Delta t$$. when being in a basin $$i$$. at an arbitrary time. As ILS does not provide statistics of these dynamics in the state space the matrix was inferred from the energy landscape using Metropolis sampling for the jumps between neighbour grid points. Following Kramer’s assumption (assuming the grid-point probability distribution within any state $$i$$ at time $$t$$. can be approximated by the steady state of state $$i$$) the transition probability from two different states ($$i,j)$$ can be calculated using the following method^57^:

2 $$T_{ij} (\Delta t) = \frac{1}{{Z_{i} \left( {3^{D} - 1} \right)}} \mathop \sum \limits_{x \in i} \mathop \sum \limits_{{\begin{array}{*{20}c} {y \in j} \\ {y\sim x} \\ \end{array} }} \min \left\{ {e^{ - \beta E\left( x \right)} ,e^{ - \beta E\left( y \right)} } \right\}$$

where $Z_{i}$ is the partition function of state $i$ given by $\mathop \sum \nolimits_{x \in i} {\text{e}}^{ - \beta E\left( x \right)}$, $D$ is number of dimensions of the landscape, $x$ and $y$ are the neighbour grid points (denoted as *y* ∼ *x*) that belong to the border of different states, $\beta = 1/k_{b} T$ representing $k_{b}$ as the Boltzmann constant and $T$ the absolute temperature of the system and finally $E\left( x \right)$ representing the energy at the grid point $x$.

The self-transition probabilities $T_{ii} (\Delta t)$ were calculated as $1 - \mathop \sum \nolimits_{i \ne j} T_{ij} (\Delta t)$. Using this method, a Markov model was constructed for each ILS 3D energy landscape for all transitions with a cut-off for saddle pair energy of < 40 kJ mol^−1^. Therefore, this model excludes very low probability transitions and very hard to reach states. As the solvent states were not crucial in the model building, they were coarse grained into a single state. Denoting the probability of a state $i$ at a time $t$ as $p_{i} \left( t \right)$, the time discrete evolution for the Markov chain can be inferred by:

3 $$p_{j} \left( {t + \Delta t} \right) = \mathop \sum \limits_{i} p_{i} \left( t \right)T_{ij} \left( {\Delta t} \right){ }$$

Iterating this Markov chain for $t \to \infty$ gives t equilibrium of the stationary probability distribution $\pi_{i} = p_{i} (\infty )$, obeying to the invariance relation $\pi_{j} = \Sigma_{i} \pi_{i} T_{ij} (\Delta t)$. The iteration process from any starting probability distribution, $p_{i} \left( 0 \right) \ne \pi_{i}$, corresponds to a relaxation process toward $\pi_{i}$, where $T_{ij} (\Delta t)$ is calculated from the above method (see Eq. 2). As the border is the same for any given states pair $ij$ the detailed balance relation $\pi_{i} T_{ij} (\Delta t) = \pi_{j} T_{ji} (\Delta t)$ is also verified. The iteration of the Markov chain (Eq. 3) utilized a probability distribution of

4 $$p_{i} \left( 0 \right) = \left\{ {\begin{array}{*{20}l} 1 \hfill & {i = solvent} \hfill \\ 0 \hfill & {i \ne solvent} \hfill \\ \end{array} } \right.$$

### Flux analysis: transition path theory

We applied transition path theory^58^ to the resultant Markov model in order to characterize the transition pathways and calculate reactive fluxes between the solvent state and the product state. Our approach is based on finding the subsets in the whole ensemble of transitions, which we can consider trajectories of molecular oxygen, leaving the solvent state (reagent) and continue until reaching the catalytic [NiFe] and [NiFeSe] centres (product states), and consider them reactive trajectories. As we cannot still pinpoint the exact place of the inactivation inside of both hydrogenases, all basins in contact (we considered the Selenium-oxygen van der Waals radius as the contact distance) with the most distant atom of the cysteines connected to the Nickel–Iron centre were considered product states and trajectories leading to those basins were considered reactive trajectories.

Using TPT the reactive trajectories were statistically characterized using committors (forward and backward). In our case the forward committor is defined as the probability that a process will reach first the product state than the solvent state, being the backwards committor the inverse. TPT also allows for the calculation of the effective flux, the net average number of reactive trajectories per time unit that transition from state $i$ to state $j$ while converging to the product states. Each basin was considered as a state and the pathways reactive trajectories. These calculations were performed using the PyEmma software^59^. Details on the use of this methodology to a similar system can be found in Damas et al*.* work^36^. Let us clarify that the effective flux aims at measuring the flux of hypothetical molecular oxygen trajectories per time unit going towards the active site. Therefore, this is a measure of the permeation of molecular oxygen towards the active site. As stated above, the flux measures trajectories per unit of time, but, contrary to other applications, the unit of time is here undefined, since we estimated the transition probabilities of the Markov model from an energy landscape (Eq. 2 above) and not from actual MD trajectories of molecular oxygen, where we could define a ∆t in the time scale of the simulations to estimate transition probabilities (*T*_*ij*_*(∆t)*). Having said this, being ∆t an undefined time, it is the same time for all processes studied here, which are based on the same potential energy function and the same methodology to estimate transition probabilities. Therefore, the fluxes can be compared between each other and between enzymes.

## Supplementary information

Supplementary information
