# Supplementary material for: Studying O2 pathways in [NiFe]- and [NiFeSe]-hydrogenases
Source: Sci Rep. 2020 Jun 29;10:10540. doi: 10.1038/s41598-020-67494-5 (PMC7324405; doi:10.1038/s41598-020-67494-5)
Supplement: Supplementary file 1 — Supplementary information [file 41598_2020_67494_MOESM1_ESM.docx]

Studying O_2_ pathways in [NiFe]- and [NiFeSe]-hydrogenases

Tiago M. Barbosa, Carla S. A. Baltazar, Davide R. Cruz, Diana Lousa and Cláudio M. Soares*

ITQB, Instituto de Tecnologia Química e Biológica António Xavier, Universidade Nova de Lisboa, Av. da República, 2780-157 Oeiras, Portugal

CORRESPONDING AUTHOR: Cláudio M. Soares

EMAIL: [claudio@itqb.unl.pt](mailto:claudio@itqb.unl.pt)

Supplementary material

# Protonation states – [NiFe] and [NiFeSe] Hydrogenases

The following table summarizes the different protonation states of the ionizable histidine residues present in the *Desulfovibrio* *gigas* [NiFe]‑Hydrogenase.

Table S1 – Histidine protonation states from Desulfovibrio gigas [NiFe] Hydrogenase. The nomenclature used is: HISA stands for a proton in the ND1 atom of the histidine, HISB for a proton in NE2 and HISH on both.

| **HIS-10 Chain S** | **HIS-42 Chain S** | | **HIS-51 Chain S** | | **HIS-58 Chain S** | | **HIS-158 Chain S** | | **HIS-190 Chain S** | | **HIS-241 Chain S** | | **HIS-12 Chain L** | | **HIS-53 Chain L** | | **HIS-66**  **Chain L** | | |
| --- | --- | --- | --- | --- | --- | --- | --- | --- | --- | --- | --- | --- | --- | --- | --- | --- | --- | --- | --- |
| **HISH** | **HISB** | | **HISH** | | **HISH** | | **HISB** | | **HISH** | | **HISA** | | **HISH** | | **HISB** | | **HISB** | | |
|  | |  | |  | |  | |  | |  | |  | |  | |  | |  |  |
| **HIS-100**  **Chain L** | **HIS-102**  **Chain L** | | **HIS-106**  **Chain L** | | **HIS-108**  **Chain L** | | **HIS-110**  **Chain L** | | **HIS-173**  **Chain L** | | **HIS-189**  **Chain L** | | **HIS-213**  **Chain L** | | **HIS-316**  **Chain L** | | **HIS-328**  **Chain L** | | |
| **HISA** | **HISB** | | **HISB** | | **HISB** | | **HISB** | | **HISB** | | **HISH** | | **HISB** | | **HISB** | | **HISB** | | |
|  | |  | |  | |  | |  | |  | |  | |  | |  | |  |  |
| **HIS-329**  **Chain L** | **HIS-343**  **Chain L** | | **HIS-377**  **Chain L** | | **HIS-462**  **Chain L** | | **HIS-519**  **Chain L** | |  | | | | | | | | | | |
| **HISB** | **HISH** | | **HISB** | | **HISB** | | **HISH** | |  |  |  |  |  |  |  |  |  |  |  |

In Table S2 the protonation states of the ionizable histidine residues of the *Desulfovibrio vulgaris* [NiFeSe]‑Hydrogenase are summarized.

Table S2 - Desulfovibrio vulgaris [NiFeSe] Hydrogenase Histidine residues protonation. The nomenclature used is: HISA stands for a proton in the ND1 atom of the histidine, HISB for a proton in NE2 and HISH on both.

| **HIS-24 Chain S** | **HIS-40 Chain S** | | **HIS-50 Chain S** | | **HIS-54 Chain S** | | **HIS-90 Chain S** | | **HIS-91 Chain S** | | **HIS-156 Chain S** | | **HIS-16 Chain L** | | **HIS-68 Chain L** | | **HIS-104 Chain L** | |
| --- | --- | --- | --- | --- | --- | --- | --- | --- | --- | --- | --- | --- | --- | --- | --- | --- | --- | --- |
| **HISB** | **HISB** | | **HISH** | | **HISB** | | **HISH** | | **HISH** | | **HISB** | | **HISH** | | **HISB** | | **HISB** | |
|  | |  | |  | |  | |  | |  | |  | |  | |  | |  |
| **HIS-107 Chain L** | **HIS-110 Chain L** | | **HIS-159 Chain L** | | **HIS-171 Chain L** | | **HIS-246 Chain L** | | **HIS-361 Chain L** | | **HIS-363 Chain L** | |  | | | | | |
| **HISB** | **HISB** | | **HISB** | | **HISB** | | **HISH** | | **HISB** | | **HISB** | |  |  |  |  |  |  |

Glutamic acid residues were all selected as charged in both structures. There were some conflicting results when comparing data from different dielectric constants in the residue GLU-294 of the [NiFeSe] Hydrogenase. As it was not possible to assert if the residue was protonated by the available data a qualitative structural analysis was performed. This consisted in aligning the [NiFeSe] and [NiFe] hydrogenases checking for homology between the residues of interest. There was homology between the GLU-294 and the [NiFeSe] hydrogenase’s GLU-276. As GLU-276 has shown clear evidence of being charged we assumed that GLU-294 would likely be also in a charged state.

# RMSD

To assess system stability, we performed RMSD analysis to both systems and both environments (with and without O2). The following figure shows the average RMSD for all hydrogenase trajectories, over time, relative to the crystal structure’s C-alphas.

#
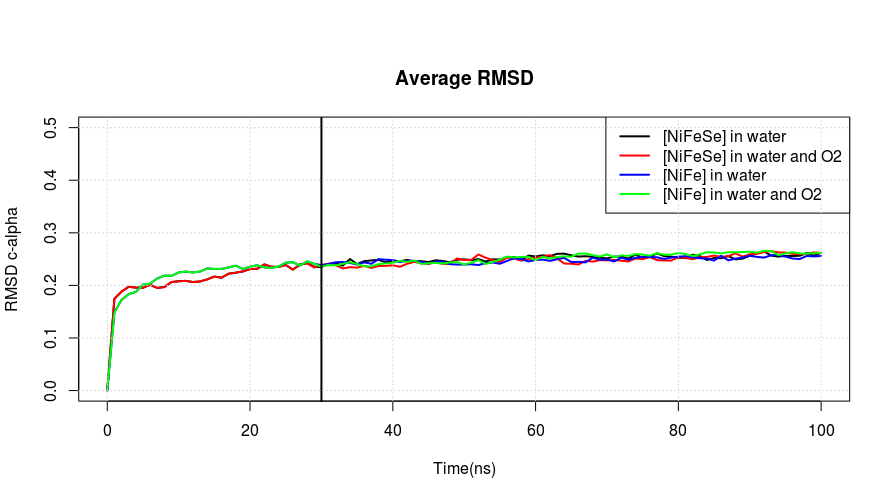


Figure S1 –C-alpha atoms RMSD averages (over the five calculated trajectories for each condition) during the simulation time(70 ns with O2 and 100 without O2) . Black line – O_2_ introduction time (30 ns).H

# Pathway residue conservation

The following table comprises the conserved and non-conserved residues (as compared with the [NiFeSe]‑hydrogenase) near the [NiFe]‑hydrogenase highest flux segments of the pathways. The corresponding residue in the [NiFeSe]‑hydrogenase is also represented. Conservation was tested using the ConSurf ^1^ server, aligning the 2frv [NiFe] and 2wpn [NiFeSe] structures with 150 homologues from UNIREF-90 using the HMMER algorithm with an e-value cut-off of 0.0001. Homologues were selected with a maximum identity percentage of 95% and minimum of 35%. Sequences were aligned using a MAAFT-INS-i method and a Bayesian evolution rate calculation method.

Table S3 – Conservation of the residues lining the highest flux zones from the [NiFe]-hydrogenase. The first column contains the residue and the chain to which it belongs. The counterpart residue by structural alignment of the [NiFeSe]-hydrogenase is placed in the second column. The third column contains the conservation status of the residue among the two hydrogenases studied here. The forth column contains the ConSurf^1^ Color Score (and not the ConSurf Conservation score).This score ranges from 1 to 9, corresponding to fully variable residue positions to fully conserved residue positions, respectively. Residues are grouped by pathway and the nomenclature is the same as the one of Table 1 in the paper.

| **[NiFe]** | **[NiFeSe]** | **Conservation status [NiFe] - [NiFeSe]** | ***ConSurf* Color Score** |
| --- | --- | --- | --- |
|  |  |  |  |
| **NF-A** | | | |
|  |  |  |  |
| ALA 52S | GLU 53S |  | **5** |
| VAL 11L | ILE 21L |  | **7** |
| SER 39L | GLY 49L |  | **6** |
| MET 357L | VAL 316L |  | **4** |
| SER 486L | SER 445L | **Conserved** | **7** |
| LEU 490L | CYS 449L |  | **7** |
| VAL 502L | VAL 461L | **Conserved** | **6** |
| PRO 517L | PRO 476L | **Conserved** | **9** |
| ILE 520L | VAL 479L |  | **7** |
| LEU 521L | ALA 480L |  | **8** |
| VAL 524L | ILE 483L |  | **7** |
| PRO 529L | PRO 488L | **Conserved** | **9** |
| CYS 530L | CYS 489L | **Conserved** | **9** |
| GLY 534L | ALA 493L |  | **8** |
| VAL 535L | VAL 494L | **Conserved** | **8** |
| **NF-B** | | | |
|  |  |  |  |
| VAL 67L | VAL 77L | **Conserved** | **9** |
| THR 69L | PRO 79L |  | **9** |
| VAL 71L | ALA 81L |  | **6** |
| HIS 72L | HIS 82L | **Conserved** | **9** |
| LEU 74L | THR 84L |  | **5** |
| MET 100L | PHE 110L |  | **2** |
| TYR 104L | TYR 114L | **Conserved** | **1** |
| ASP 107L | SER 117L |  | **9** |
| LEU 115L | LEU 125L | **Conserved** | **9** |
| ASN 460L | GLU 419L |  | **8** |
| ALA 461L | ALA 420L | **Conserved** | **8** |
| PRO 462L | PRO 421L | **Conserved** | **9** |
| ARG 463L | ARG 422L | **Conserved** | **9** |
| LEU 466L | LEU 425L | **Conserved** | **9** |
| **NF-C** | | | |
|  |  |  |  |
| THR 61L | VAL 61L |  | **5** |
| GLN 62L | GLN 72L | **Conserved** | **8** |
| ARG 63L | ARG 73L | **Conserved** | **9** |
| ALA 64L | ILE 74L |  | **7** |
| CYS 65L | CYS 75L | **Conserved** | **9** |
| CYS 68L | CYS 78L | **Conserved** | **9** |
| THR 69L | PRO 79L |  | **9** |
| TYR 70L | THR 80L | **Conserved** | **4** |
| HIS 72L | HIS 82L | **Conserved** | **9** |
| HIS 468L | HIS 427L | **Conserved** | **9** |
| VAL 484L | SER 444L |  | **9** |

The following table comprises the conserved and non-conserved residues (as compared with the [NiFe]‑hydrogenase) near the [NiFeSe]‑hydrogenase highest flux segments of the pathways. The corresponding residue in the [NiFe]‑hydrogenase is also represented as well as the ConSurf score.

Table S4 - Conservation of the residues lining the highest flux zones from the [NiFeSe]-hydrogenase. The first column contains the residue and the chain to which it belongs. The counterpart residue by structural alignment of the [NiFe]-hydrogenase is placed in the second column. The third column contains the conservation status of the residue among the two hydrogenases studied here. The forth column contains the ConSurf^1^ Color Score (and not the ConSurf Conservation score).This score ranges from 1 to 9, corresponding to fully variable residue positions to fully conserved residue positions, respectively. Residues are grouped by pathway and the nomenclature is the same as the one of Table 1 in the paper.

| **[NiFeSe]** | **[NiFe]** | **Conservation status [NiFeSe-NiFe]** | ***ConSurf* score** |
| --- | --- | --- | --- |
| **NFS-A** | | | |
|  |  |  |  |
| LEU 13S | LEU 12S | **Conserved** | **7** |
| GLY 17S | GLU 16S |  | **7** |
| CYS 18S | CYS 17S | **Conserved** | **9** |
| GLU 28L | GLU 18L | **Conserved** | **9** |
| ARG 53L | ARG 43L | **Conserved** | **9** |
| GLN 72L | GLN 62L | **Conserved** | **8** |
| ILE 74L | ALA 64L |  | **9** |
| CYS 75L | CYS 65L | **Conserved** | **9** |
| GLY 76L | GLY 66L | **Conserved** | **9** |
| PRO 79L | THR 69L |  | **9** |
| PRO 184L | PRO 218L | **Conserved** | **9** |
| HIS 185L | HIS 219L | **Conserved** | **9** |
| GLY 491L | ALA 532L |  | **9** |
| **NFS-B** | | | |
|  |  |  |  |
| VAL 77L | VAL 67L | **Conserved** | **9** |
| ASN 113L | GLN 103L |  | **9** |
| GLN 116L | HIS 106L |  | **9** |
| SER 117L | ASP 107L |  | **9** |
| LEU 120L | VAL 110L |  | **9** |
| HIS 121L | HIS 111L | **Conserved** | **8** |
| HIS 124L | HIS 114L | **Conserved** | **7** |
| TYR 162L | TYR 196L | **Conserved** | **9** |
| ALA 165L | ALA 199L | **Conserved** | **8** |
| LEU 166L | LEU 200L | **Conserved** | **6** |
| ARG 169L | GLN 203L |  | **9** |
| PRO 421L | PRO 462L | **Conserved** | **6** |
| ARG 422L | ARG 463L | **Conserved** | **9** |
| **NFS-C** | | | |
|  |  |  |  |
| PHE 45S | TYR 44S |  | **8** |
| ILE 58L | ILE 48L | **Conserved** | **8** |
| ARG 62L | ARG 52L | **Conserved** | **9** |
| ILE 70L | PHE 60L |  | **5** |
| VAL 71L | THR 61L |  | **8** |
| ILE 74L | ALA 64L |  | **9** |
| CYS 492L | CYS 533L | **Conserved** | **9** |
| HIS 495L | HIS 536L | **Conserved** | **9** |

# References

1. Ashkenazy, H. *et al.* ConSurf 2016: an improved methodology to estimate and visualize evolutionary conservation in macromolecules. *Nucleic Acids Res.* **44**, W344–W350 (2016).
